# A receptor kinase complex refines cambium activity in *Arabidopsis*

**DOI:** 10.1073/pnas.2532481123

**Published:** 2026-06-22

**Authors:** Qing He, Hanan Alhowty, Prodeep Paudel, Xixi Zhang, Wenbin Wei, Tuomas Sipilä, Ehmke Pohl, Ari Pekka Mähönen, Ville O. Paavilainen, Raymond Wightman, Yuan Qin, J. Peter Etchells

**Affiliations:** ^a^ Department of Biosciences, Durham University, Durham DH1 3LE, United Kingdom; ^b^ College of Life Sciences, Fujian Provincial Key Laboratory of Haixia Applied Plant Systems Biology, Haixia Institute of Science and Technology, Fujian Agriculture and Forestry University, Fuzhou 350002, China; ^c^ Department of Biology, Jazan University, Jazan 82817, Saudi Arabia; ^d^ Department of Organismal and Evolutionary Biology, Faculty of Biological and Environmental Sciences and Viikki Plant Science Centre, University of Helsinki, Helsinki 00014, Finland; ^e^ Microscopy Core Facility, Sainsbury Laboratory, University of Cambridge, Cambridge CB2 1LR, United Kingdom

**Keywords:** receptor kinase, *Arabidopsis*, development, meristem, vascular development

## Abstract

In plant development, receptor kinases are often active in disparate cell types, with each requiring vastly different signaling outputs. The ERECTA (ER) receptor kinase and its homologs Erecta-Like1 (ERL1) and ERL2 exemplify this pleiotropy. In *Arabidopsis*, they influence stomatal patterning, shoot meristem function, and vascular cambium activity, among other functions. Such diverse functionality raises the question of how ER signaling can specify distinct cell behaviors. One key mechanism occurs via cell-type specific interactions with coreceptors, ligands, or other proteins that modulate signaling. However, little is known about ER interactors in the vascular cambium, a bifacial stem cell niche that generates phloem and xylem. Combinatorial mutations between *ER*, *ERL1,* and *ERL2* and receptor kinases of a second family, Phloem Intercalated with Xylem (*PXY)*, *PXY-LIKE1* (*PXL1)*, and *PXL2*, show severe cambial defects, but the mechanism underpinning these phenotypes is not known. Here, we show that PXY and PXL proteins form protein complexes with ER family members. In genetic analyses, plant lines in which PXY signaling was constitutively active had dramatic phenotypic changes that required the presence of ER or ERL2. Our results demonstrate that PXY signaling mediated cambium regulation in part depends on ER signaling and explains ER function in the cambium. Because the cambium produces xylem, which constitutes the wood in vascular plants, our findings position PXY–ER complexes at the center of the accumulation of this versatile biomaterial and essential carbon sink.

## Significance

The largest reservoir of terrestrial biomass is the wood within plant stems. Consisting of xylem cells, wood is derived from the cambium, a stem cell population that is maintained by non-cell autonomous signaling. The central component of this system is PXY, a receptor protein present on the plasma membrane of cambium cells. Here, we show that PXY protein interacts with a second membrane-localized receptor, ER, to promote cambium fate, suggesting that this receptor protein complex acts at the center of plant biomass deposition. While receptors are widely described as interacting with coreceptors in plants, far fewer receptor–receptor interactions are known. We propose that similar such plant receptor–receptor complexes may play wide-ranging roles in specification of signaling specificity and crosstalk.

Wood is a globally significant renewable biomaterial comprised of xylem cells. It forms on one side of the cambium, a bifacial stem cell niche, which also produces phloem on its opposing side. Maintenance of stem cells within the cambium is crucial to continuous xylem and phloem production. Non-cell autonomous signaling is central to cambium stem cell maintenance. Here, CLE41 and CLE44 are expressed in the phloem and are processed to release a dodecapeptide peptide ligand, Tracheary Element Differentiation Inhibitory Factor (TDIF). TDIF forms a gradient that peaks in the phloem and tapers off through the cambium toward the xylem. As TDIF diffuses from the phloem, it is perceived and sequestered by a plasma membrane-localized receptor kinase, PXY. PXY has an expression peak at the xylem–cambium boundary which also tapers through the cambium (1–6). TDIF–PXY complexes activate expression of four AIL-family transcription factors, *ANT*, *AIL5*, *AIL6*, and *AIL7* (cambium-expressed *AILs*; *CAILs*) which define the cambium stem cell niche and are essential for cambium function (2). In addition to the CAILs, TDIF–PXY signaling also regulates the expression of a suite of further transcription factor targets. BES1 promotes xylem differentiation by repressing cambium activity, where it acts as a negative regulator of *WOX4*. Active TDIF–PXY marks BES1 for degradation, releasing WOX4 to activate cambium cell division (7–9). A further three transcription factors, *WOX14*, *TMO6*, and *LBD4* constitute a coherent feedforward loop that is TDIF–PXY activated and contributes to cambium homeostasis (10).

PXY is likely to function redundantly with other factors at the plasma membrane. *pxy* single mutants retain a degree of radial growth; plants are characterized by hypocotyls with fewer, larger cells in transverse section and disordered vascular tissue, and although *pxy* phenotypes are enhanced when its two homologues, *pxl1* and *pxl2* are also mutated, this enhancement is relatively mild (1, 11). However, the original *pxy* allele, isolated in the L*er* background, had a stronger phenotype characterized by a greater disruption to vascular organization than null alleles in the Col-0 background. L*er* carries a mutation in the *ER* gene, which encodes a member of a second family of receptor kinases. The *er* mutation was subsequently shown to be an enhancer of the *pxy* phenotype. Indeed, when members of the ER family are mutated in combination with PXY family members, more severe phenotypes are observed (11, 12). As such, *er* is a strong enhancer of *pxy*, implicating it in cambium development.

ER and its homologues are expressed uniformly across xylem, cambium, and phloem (12, 13), but ER functions in multiple additional developmental pathways. It regulates stomatal spacing, shoot elongation, inflorescence architecture, xylem differentiation, and female germline specification (13–17), among others. How ER generates cell-type specific outputs given its pleiotropy represents an interesting problem, and in some tissues, the answer lines in the cell-type specific complexes that it forms. For example, ER forms a complex with a second receptor protein, Too Many Mouths (TMM) which allows it to bind ligands EPF1 and EPF2 in regulation of stomatal patterning. By contrast, ER binds the EPFL4, EPFL5, and EPFL6 ligands independently of TMM in regulation of shoot elongation (18–23). However, how ER performs its cambium-specific function is not known.

Here, we show that despite broad expression across vascular tissues (12, 13), *ER* is specifically required within the domain of *PXY* expression for cambium activity. Thus, ER is required within the cambium itself, and it does not exert its influence over cambium homeostasis from adjacent tissues. This led to the possibility that ER and its homologues might form protein complexes with PXY, PXL1, and PXL2 to control cambium development. Protein interaction assays demonstrated that this was indeed the case and combined with genetic experiments, evidence shows that ER and ERL2 have a greater role in cambium homeostasis than ERL1. Further experiments, where phenotypes of lines in which TDIF–PXY signaling was constitutively active were attenuated by loss of *ER*, demonstrate that ER is required for some TDIF–PXY signaling outputs. Thus, PXY-mediated regulation of cambium activity likely occurs via the formation of complexes with ER at the plasma membrane. As such, PXY–ER heteromer formation underpins wood production and thus the formation of a significant proportion of terrestrial biomass.

## Results

### *PXY* and *ER* Interact in the Cambium.

**Fig. 1.**
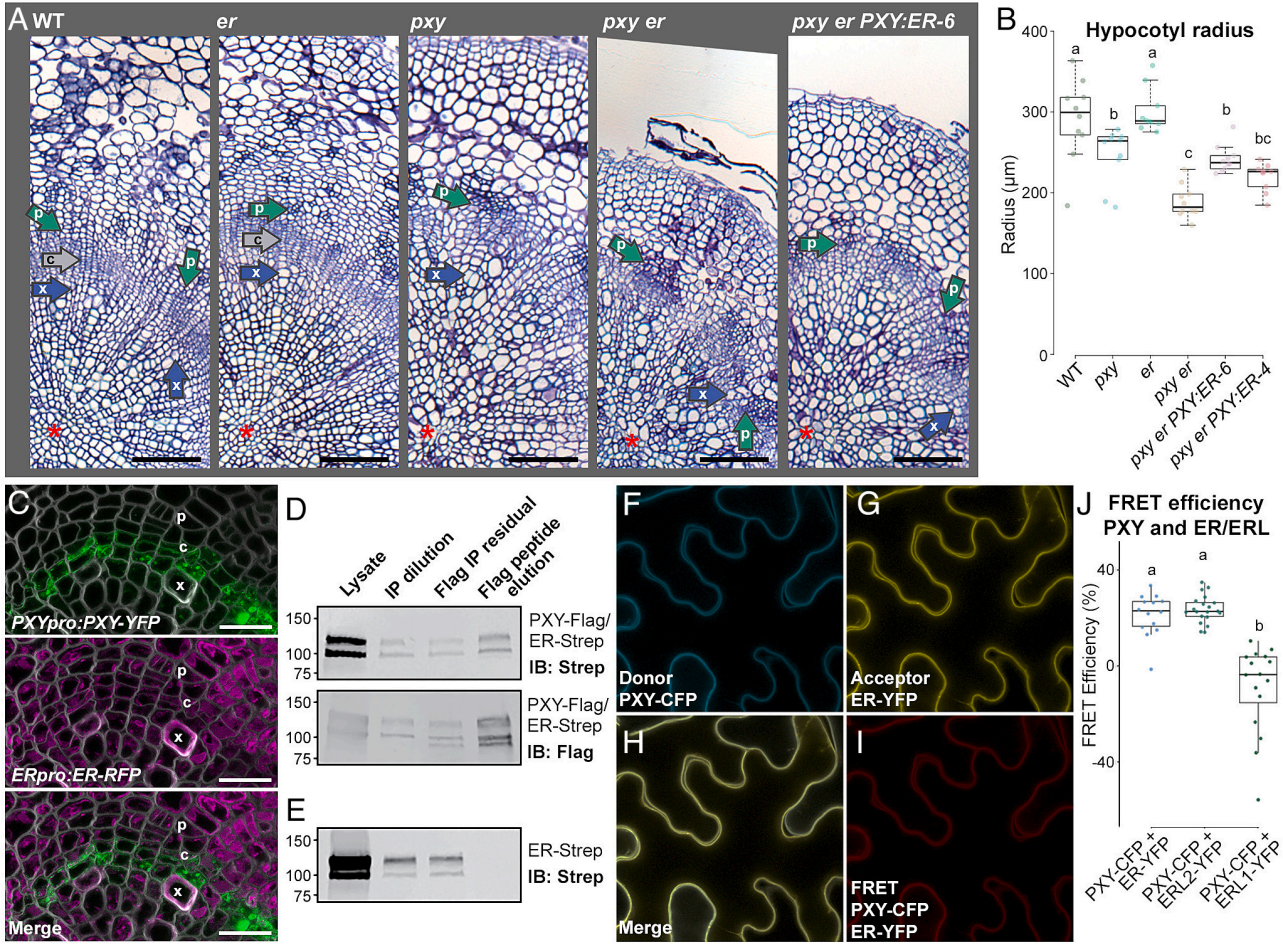
ER is required within the domain of *PXY* expression for cambium activity. (*A*) Transverse sections of wild type, *er*, *pxy*, *pxy er*, and *pxy er PXYpro:ER* hypocotyls at 4 wk, showing that *ER* expressed from the PXY promoter partially suppresses *pxy er* secondary growth defects. (*B*) boxplot showing radial growth in wild type, *er*, *pxy*, *pxy er*, and *pxy er PXY:ER* hypocotyls. (*C*) Colocalization of PXY-YFP and ER-RFP in root transverse sections. (*D* and *E*) Co-IP of PXY and ERECTA in absence of other plant proteins. (*D*) Twinstrep-tag and Flag-tag immunoblot of flag Immunoprecipitation from PXY-Flag/ERECTA-Twinstrep transfected sf21 insect cell membrane lysate (*E*) Twinstrep-tag immunoblot of ERECTA transfected sf21 insect cell membrane lysate (n = 3; representative images shown. (*F*–*I*) Localization of PXY-CFP (*F*), ER-YFP (*G*), and overlap (*H*) in *Nicotiana* epidermal cells. (*I*) FRET signal. (*J*) Graph showing FRET efficiencies of PXY-CFP and ER/ERL1/ERL2-YFP pairs. Scales are 50 µM in (*A*) and 20 µM in (*B*); red asterisks mark the hypocotyl center; “p” indicates phloem, “c” marks the cambium, and “x” the xylem. Letters marked on boxplots in (*B* and *J*) denote homogeneous subsets (ANOVA plus Tukey).

It has long been known that TDIF–PXY signaling is a key regulator of vascular development and that cambium initiation and maintenance are perturbed in its absence (1, 3, 6), exemplified by reductions in cambial cell division in *pxy* mutants (1, 3). These phenotypes are significantly enhanced by loss-of-function mutations in *ER* (11, 12). ER has a pleiotropic function; thus, the enhancement of *pxy* by *er* could be due to regulation of cambium activity by ER in combination with PXY. Alternatively, a secondary effect in *er* mutants with already perturbed growth could be responsible for the increased severity of *pxy er* over *pxy*. To discriminate between these possibilities, we generated a construct, *PXYpro:ER*, where *ER* was expressed under the control of the *PXY* promoter that was transformed into *pxy er* double mutants. If the enhancement of *pxy* by *er* was due to pleiotropy, expression of *ER* only in the *PXY* domain would have little effect on the *pxy er* phenotype, and *pxy er PXYpro:ER* plants would be reminiscent of *pxy er*. By contrast, if ER acts within the cambium itself to control cambium function, *pxy er PXYpro:ER* lines would have a phenotype closer to that of *pxy* single mutants. A range of phenotypes in independent *pxy er PXYpro:ER* lines was obtained, including those with radial growth levels between that of *pxy* and *pxy er* lines, or with attenuation of the *pxy er* radial growth phenotype, to the level observed in *pxy* single mutants (Fig. 1 *A* and *B*). This demonstrates that PXY and ER coregulate radial growth within the *PXY* expression domain.

As partial suppression of *pxy er* by *PXYpro:ER* demonstrated that ER is required within the domain of *PXY* expression for cambium function, we next addressed the molecular mechanism underlying the genetic interaction between *PXY* and *ER.* A high-throughput Extracellular Interactome Assay (EICA) dataset which identified putative extracellular interactions between *Arabidopsis* receptor kinases, included interactions between the LRR domains of ER and PXY, ER and PXL1, and PXL2 and ERL2, albeit at low confidence levels (24, 25). We thus hypothesized that genetic interactions between *PXY* and *ER* family members might be underpinned by protein complex formation between these two receptor kinases. Previous reports indicate that *ER* is broadly expressed across xylem, cambium, and phloem. In contrast, *PXY* expression is most intense in differentiating xylem and tapers off toward the cambium stem cells (2–4, 13, 26, 27). In support of these observations, expression of *PXYpro:PXY-YFP* and *ERpro:ER-RFP* constructs were observed to overlap in transverse hypocotyl sections. As such, protein interactions between PXY and ER could occur in the overlapping domain (Fig. 1*C*). To test this possibility, PXY-FLAG and ER-STREP were coexpressed in Sf21 insect cells using the baculovirus expression system. PXY-FLAG and ER-STREP were copurified as a stable complex from the Sf21 cell membranes (Fig. 1 *D* and *E*), indicating that PXY and ER can interact without additional plant proteins. Next, Förster Resonance Energy Transfer (FRET), which relies on the proximity of fluorophores with FRET being detected if two fluorophore-tagged proteins interact was used to test this possibility in plant tissues. *35S:PXY-CFP* and *35S::ER-YFP* constructs were generated and coinfiltrated into *Nicotiana* leaves. Epidermal cells expressing fluorescently labelled PXY and ER were analyzed for colocalization of signal and FRET. A YFP signal was observed upon excitation of CFP (Fig. 1 *F*–*I*), and energy transfer was measured at 21.4%, indicating PXY and ER proteins interact (Fig. 1*J*). We then determined if PXY could also complex with ERL1 and ERL2 by generating *35S::ERL1-YFP* and *35S::ERL2-YFP* constructs and coinfiltrating them with *35S:PXY-CFP*. A FRET signal was observed between PXY-CFP and ERL2-YFP which was measured with an efficiency of 21.8%. By contrast, we found no evidence for interactions between PXY and ERL1 (Fig. 1*G* and *SI Appendix*, Fig. S1 *A* and *B*). To strengthen the evidence for PXY protein–protein interactions with members of the ER family, Coimmunoprecipitation (Co-IP) was performed following generation of FLAG and HA epitope-tagged protein constructs. A *35S:PXY-FLAG* translational fusion construct was coinfiltrated into *Nicotiana* leaves with *35S:ER-HA* or *35S:ERL2-HA*. PXY-FLAG was detected in pull downs using anti-HA beads, but not in *35S:PXY-FLAG* only controls. Similarly, when anti-FLAG beads were used ER-HA or ERL2-HA were pulled when *35S:PXY-FLAG* was coinfiltrated with *35S:ER-HA* or *35S:ERL2-HA,* but not in *35S:ER-HA* or *35S:ERL2-HA* only controls (*SI Appendix*, Fig. S1 *C* and *D*). Thus, PXY interacts with ER and ERL2 in plant cells.

### Protein Complexes Form between Members of the PXY and ER Families.

**Fig. 2.**
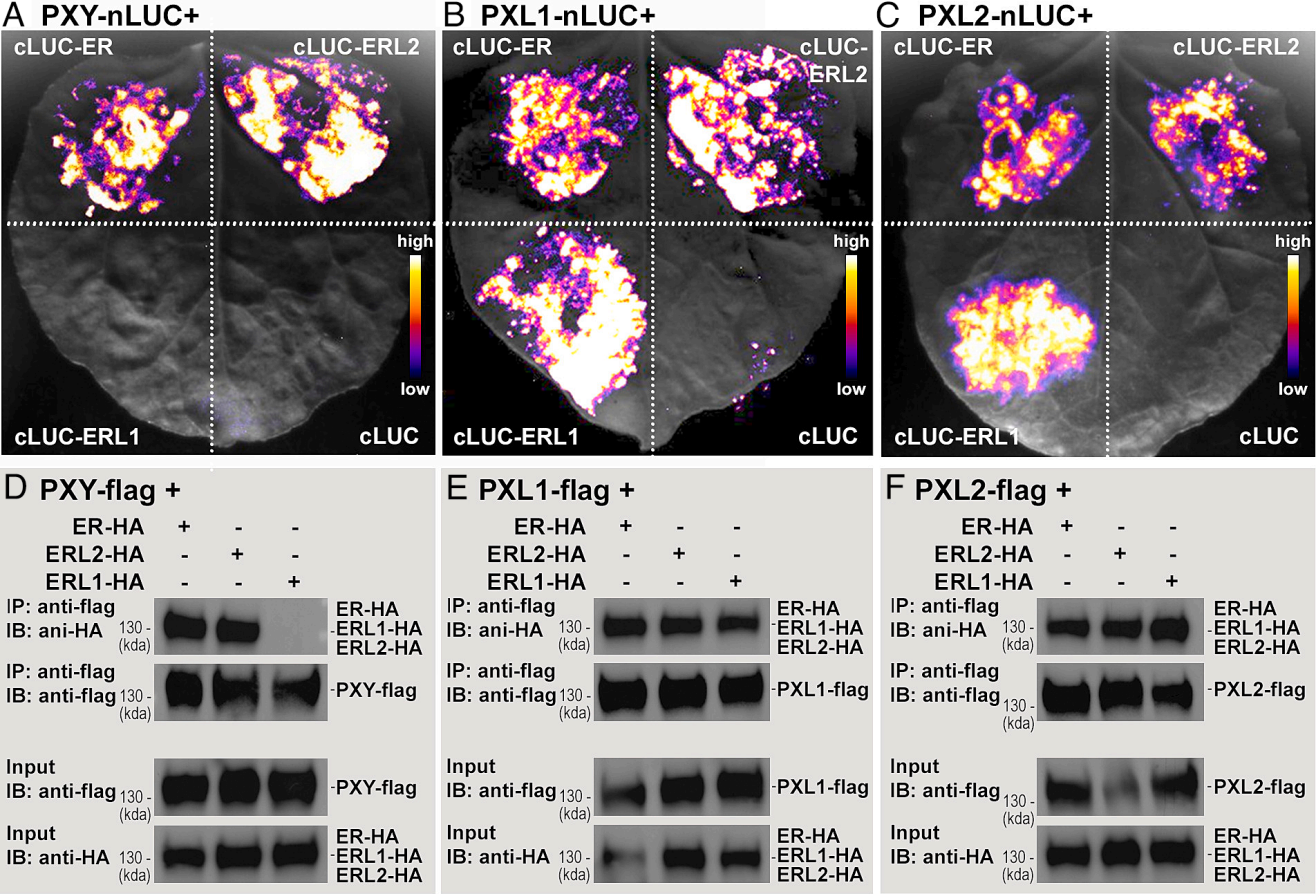
Members of the PXY and ER families form protein complexes. (*A*–*C*) Split luciferase assays in *Nicotiana* leaves in which the n-term of Luciferase was fused to PXY (*A*), PXL1 (*B*), and PXL2 (*C*) and was coinfiltrated with the c-term alone, or the c-term fused to ER, ERL1, or ERL2. (*D*–*F*) Co-IP of ER-HA, ERL1-HA, or ERL2-HA with PXY-flag (*D*), PXL1-flag (*E*), or PXL2-flag (*F*). Immunoblot (IB); Immunoprecipitation (IP).

We sought to both expand our analysis to test protein interactions between PXL1 and PXL2, and members of the ER family, and confirm that PXY does indeed not interact with ERL1 (as we did not test whether PXY coimmunoprecipitated with ERL1 in *Nicotiana* leaves as no evidence of an interaction had been obtained in FRET). Thus, further protein interaction assays were performed using a split luciferase (LUC) system. Here, the N-terminal part of LUC was fused to PXY (*35S:PXY-nLUC*), and the C-terminal part to either ER (*35S:ER-cLUC*), ERL1, or ERL2. The C-terminus alone was also used as a negative control. *Nicotiana* leaves were coinfiltrated with Agrobacterium carrying *35S:PXY-nLUC* and either *35S:ER-cLUC*, *35S:ERL1-cLUC*, *35S:ERL2-cLUC*, or *35S:cLUC*. Upon luciferin application, a signal was detected for *PXY-nLUC*/*ER-cLUC* and *PXY-nLUC*/*ERL2-cLUC* pairs, but not for *PXY-nLUC*/*ERL1-cLUC* and *PXY-nLUC*/*cLUC* pairs (Fig. 2*A*). These results provided further support for the formation of PXY–ER and PXY–ERL2 complexes and confirmed that PXY and ERL1 did not interact. By contrast, in similar assays in which *PXY–nLUC* was replaced with either *PXL1–nLUC* (Fig. 2*B*) or *PXL2-nLUC* (Fig. 2*C*), luciferase signal, indicating complex formation, was observed between PXL1/2 and ER/ERL1/ERL2.

While EICA, Sf21 cell, FRET, split Luciferase, and *Nicotiana* Co-IP experiments supported protein interactions between PXY and ER/ERL2 and between PXL1/PXL2 and ER/ERL1/ERL2, all these methods used either in vitro or heterologous systems. Consequently, *Arabidopsis* cell suspension cultures were used to determine whether PXY–ER or PXY–ERL2 complexes form in *Arabidopsis* cells. PXY–ERL1 pairs were used as a negative control. *35S:ER-HA*, *35S:ERL1-HA*, and *35S:ERL2-HA* constructs were transformed into *Arabidopsis* protoplasts as pairs with *35S:PXY-FLAG*. ER-HA or ERL2-HA were detected in the presence of PXY-FLAG when protein extracts were coincubated with anti-FLAG beads. Furthermore, PXY-FLAG was detected in the presence of ER-HA or ERL2-HA, when pulled down using anti-HA beads (Fig. 2 *D*–*F*). PXL1 pulled down ER, ERL1, and ERL2, as did PXL2. Thus, PXY forms complexes with ER and ERL2, while PXL1 and PXL2 form complexes with all three members of the ER family in *Arabidopsis*. These observations were confirmed in reciprocal Co-IPs where HA-tagged members of the ER family pulled down flag-tagged PXY family members, with the exception of the PXY ERL1 pair (*SI Appendix*, Fig. S2).

**Fig. 3.**
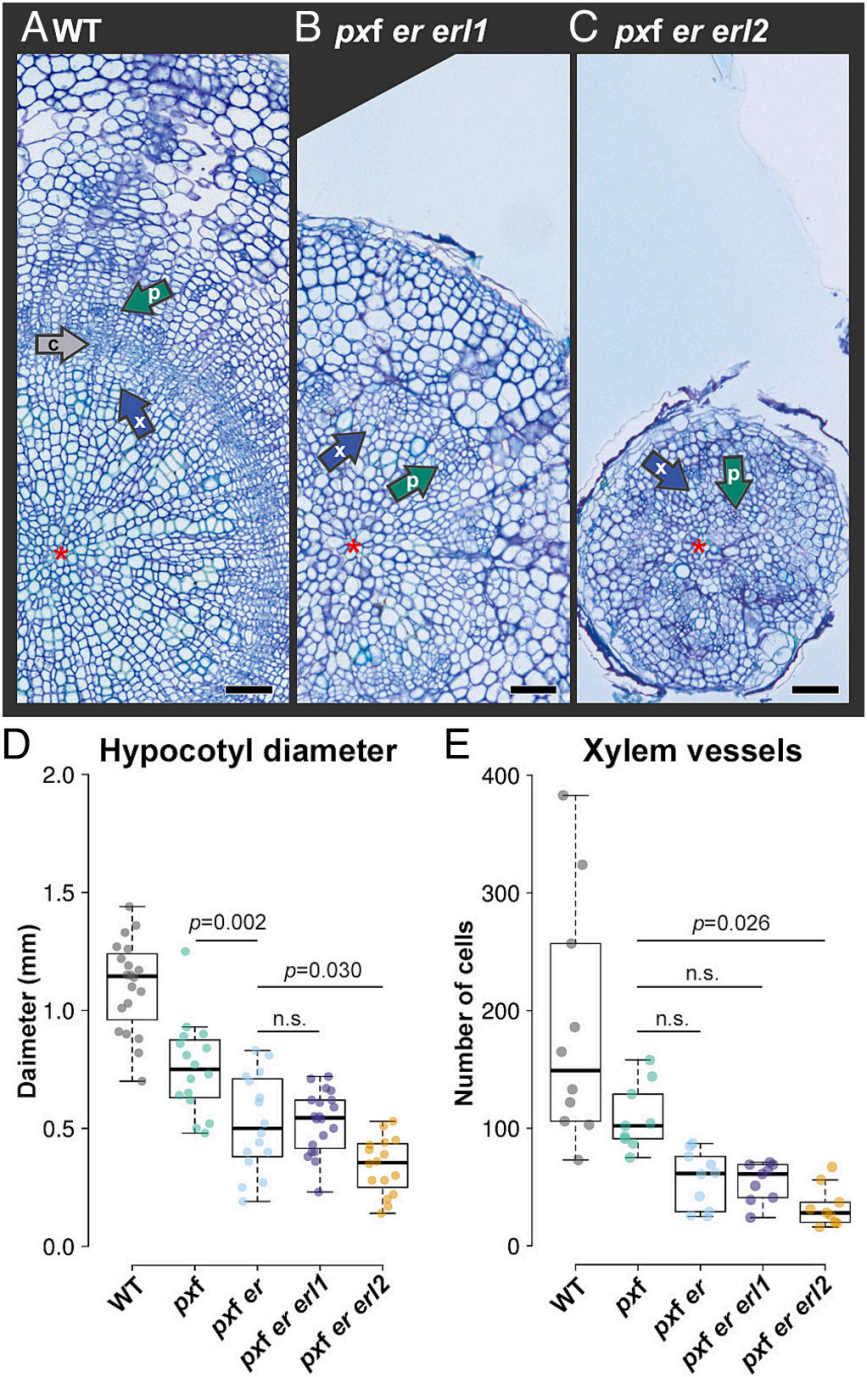
Loss of *pxy/l* genes and *er* is enhanced by *erl2* but not *erl1.* (*A*–*C*) Transverse sections of 4-wk-old hypocotyls (*px*f, for *pxy* family, denotes *pxy pxl1 pxl2* triple mutants). Wild type (*A*), *px*f *er erl1* (*B*), and *px*f *er erl2* (*C*) sections are shown. (*D* and *E*) Boxplots showing hypocotyl diameter (*D*) and number of xylem vessels (*E*). Red asterisks mark the central xylem axis; the green arrow denotes differentiated phloem; the blue arrow is differentiated xylem; the gray arrow marks the cambium. Lines above boxes in (*D* and *E*) show significant differences (ANOVA + Tukey).

To further investigate ERL1’s inability to interact with PXY, we tested whether PXY and ERL1 might interact with chimeric ER/ERL1 or PXY/PXY-like proteins, respectively, using the split Luciferase system. Here, PXY did not interact with a chimera constituted of the ERL1 extracellular domain and the ER cytoplasmic domain. Conversely, ERL1 bound a protein consisting of the PXY extracellular domain and PXL1 cytoplasmic domain. As such, we could identify no clear domain-specific pattern that defined the lack of PXY–ERL1 binding (*SI Appendix*, Fig. S3). Nevertheless, to determine the potential implications of the inability of ERL1 to interact with PXY on cambium activity, *pxy pxl1 pxl2 er erl1* plants were compared to *pxy pxl1 pxl2 er erl2* lines alongside parental controls (Fig. 3 *A*–*C*). Radial growth was measured, and *pxy pxl1 pxl2 er erl2* hypocotyls demonstrated a significant reduction in radial growth compared to *pxy pxl1 pxl2 er* controls, whereas *pxy pxl1 pxl2 er erl1* plants demonstrated no difference (Fig. 3*D*). Xylem vessel numbers (as a representative of cells derived from the cambium) were also determined in hypocotyls. *pxy pxl1 pxl2 er erl1* plants demonstrated no difference in xylem vessel number compared to *pxy pxl1 pxl2* and *pxy pxl1 pxl2 er* controls. By contrast, *pxy pxl1 pxl2 er erl2* plants demonstrated a significant reduction in xylem vessel number (Fig. 3*E*). Thus, genetic analysis demonstrated that ERL1 has less ability to influence cambium activity than ERL2. Although ERL1 and ERL2 may function differently in a PXY-independent manner, given that PXY itself has the greater effect on vascular development than PXL1 and PXL2 (1), and that PXY and ERL1 do not interact, one explanation for the weaker *pxy pxl1 pxl2 er erl1* phenotype is ERL1’s failure to complex with PXY.

### Loss of ER Attenuates PXY Signaling.

**Fig. 4.**
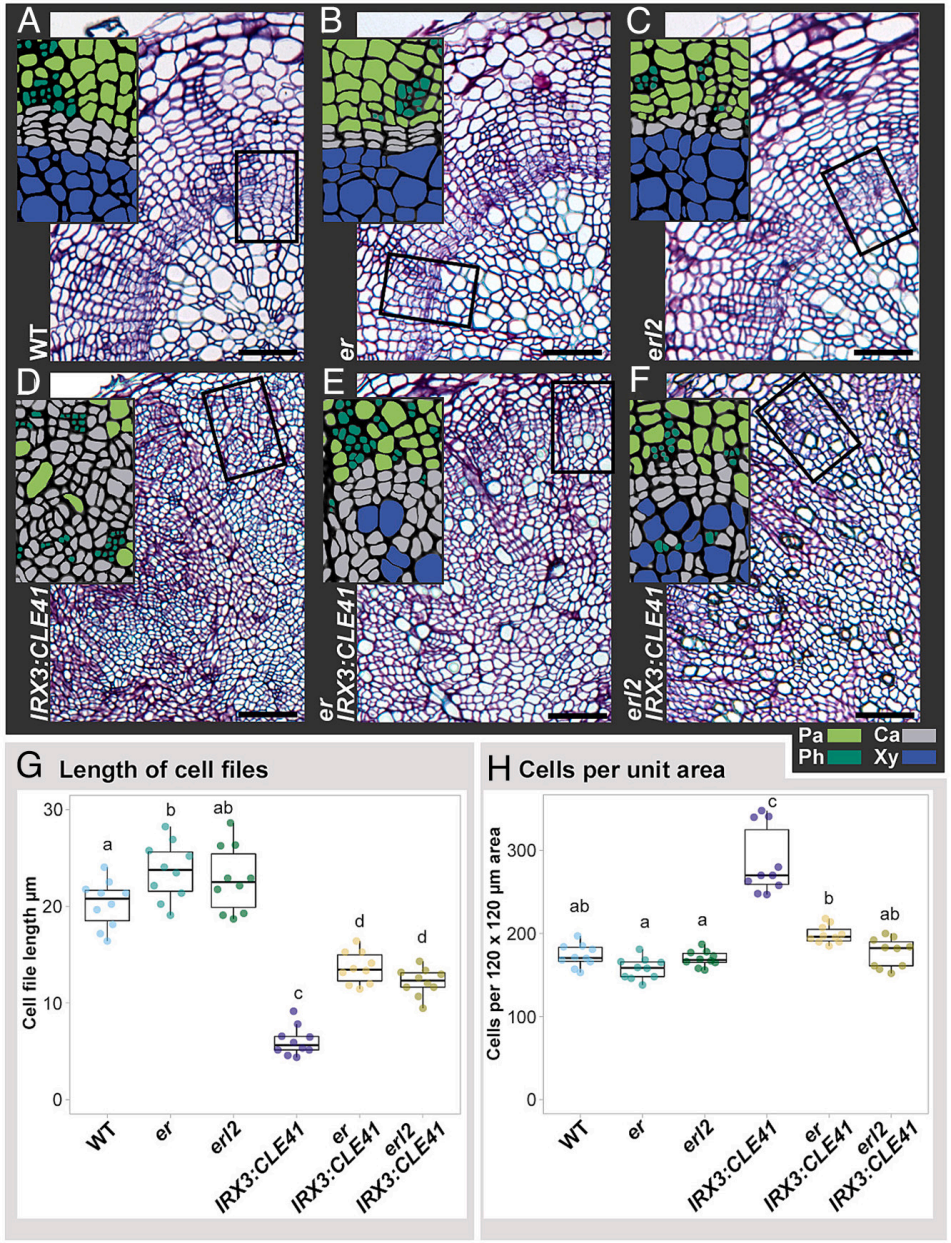
*er* and *erl2* suppress TDIF overproduction phenotypes. (*A*–*F*) Transverse sections of hypocotyls. Thin sections show the extent of radial growth. Black rectangles mark the area of cambium shown in diagrammatic representations on the left. Xylem and phloem are separated by cambium in wild type (*A*), *er* (*B*), and *erl2* (*C*). In *IRX3:CLE41* lines (*D*), very few xylem cells are apparent, and the cambium is disordered. Organization of a cambial zone is restored in *er IRX3:CLE41* (*E*) and *erl2 IRX3:CLE41* (*F*) lines. (*G*) Boxplot showing length of cell files running parallel to the radial axis of the stem. (*H*) Boxplot showing cells per 14,400 µM^2^ in hypocotyl transverse sections. Letter above boxes mark significance groups (*G* and *H*; ANOVA + Tukey). Scales are 50 µM; Pa is parenchyma, Ca cambium, Ph phloem, and Xy xylem.

To better understand the influence of ER and ERL2 on vascular development in the context of TDIF–PXY signaling, we sought to perturb ER and ERL2 signaling in a background in which PXY signaling was constitutively active. TDIF, the peptide ligand that binds to PXY, PXL1, and PXL2 (3, 28), is derived from *CLE41* and *CLE44* which are phloem-expressed (3, 5, 6). Expressing *CLE41* from a xylem-specific promoter results in ectopic PXY activation (6). The *IRX3* promoter drives strong expression in differentiating xylem (29). In wild-type hypocotyls, cambial cell divisions are present in a narrow ring of tissue between xylem and phloem. Predominantly periclinal in nature, these cell divisions give rise to long cell files that align with the hypocotyl radial axis. By contrast, ectopic cambium with misaligned cell divisions are apparent throughout the hypocotyl in *IRX3:CLE41* (6) where this misalignment of cell division leads to very short cell files (Fig. 4 *A*, *D*, and *G*).

We tested whether ER and ERL2 were required for *IRX3:CLE41* phenotypes by crossing *er* and *erl2* mutants into an *IRX3:CLE41* background. The resulting *er IRX3:CLE41* and *erl2 IRX3:CLE41* plants were compared to controls. By contrast to *IRX3:CLE41* hypocotyls, aligned cell divisions in a defined cambial zone were present in those from *er IRX3:CLE41* and *erl2 IRX3:CLE41* in a position comparable to the cambial zone of wild-type plants (Fig. 4 *E*–*G*). Furthermore, average cell file length more than doubled in *er IRX3:CLE41* and *erl2 IRX3:CLE41* relative to *IRX3:CLE41* (Fig. 4 *E*–*G*), consistent with partial restoration of a spatially restricted cambium. Dividing cambium cells are typically smaller in size when compared to xylem vessels and parenchyma, which occupy the center of wild-type hypocotyls. Consequently, an increase in cell size might be expected concomitant with restoration of spatially restricted cambium in *er IRX3:CLE41* and *erl2 IRX3:CLE41* lines, relative to *IRX3:CLE41* where the tissue was occupied predominantly with small cells reminiscent of actively dividing cambia. Indeed, cells in the center of the hypocotyl of *er IRX3:CLE41* and *erl2 IRX3:CLE41* lines were larger than those observed in *IRX3:CLE41* lines. The number of cells per unit area was reduced by 31% in *er IRX3:CLE41*, and by 38% *erl2 IRX3:CLE41* when compared to *IRX3:CLE41* (Fig. 4*H*). Subsequently, we tested whether *erl1* could also partially suppress *IRX3:CLE41*, but *IRX3:CLE41* and *erl1 IRX3:CLE41* plants were indistinguishable (*SI Appendix*, Fig. S4). *er* and *erl2* thus partially suppressed *IRX3:CLE41* phenotypes, but *erl1* did not. This confirmed the importance of functional ER and ERL2 signaling (but not ERL1) for fully active TDIF–PXY signaling.

### ER Contributes to Regulation of TDIF–PXY Transcriptional Targets.

**Fig. 5.**
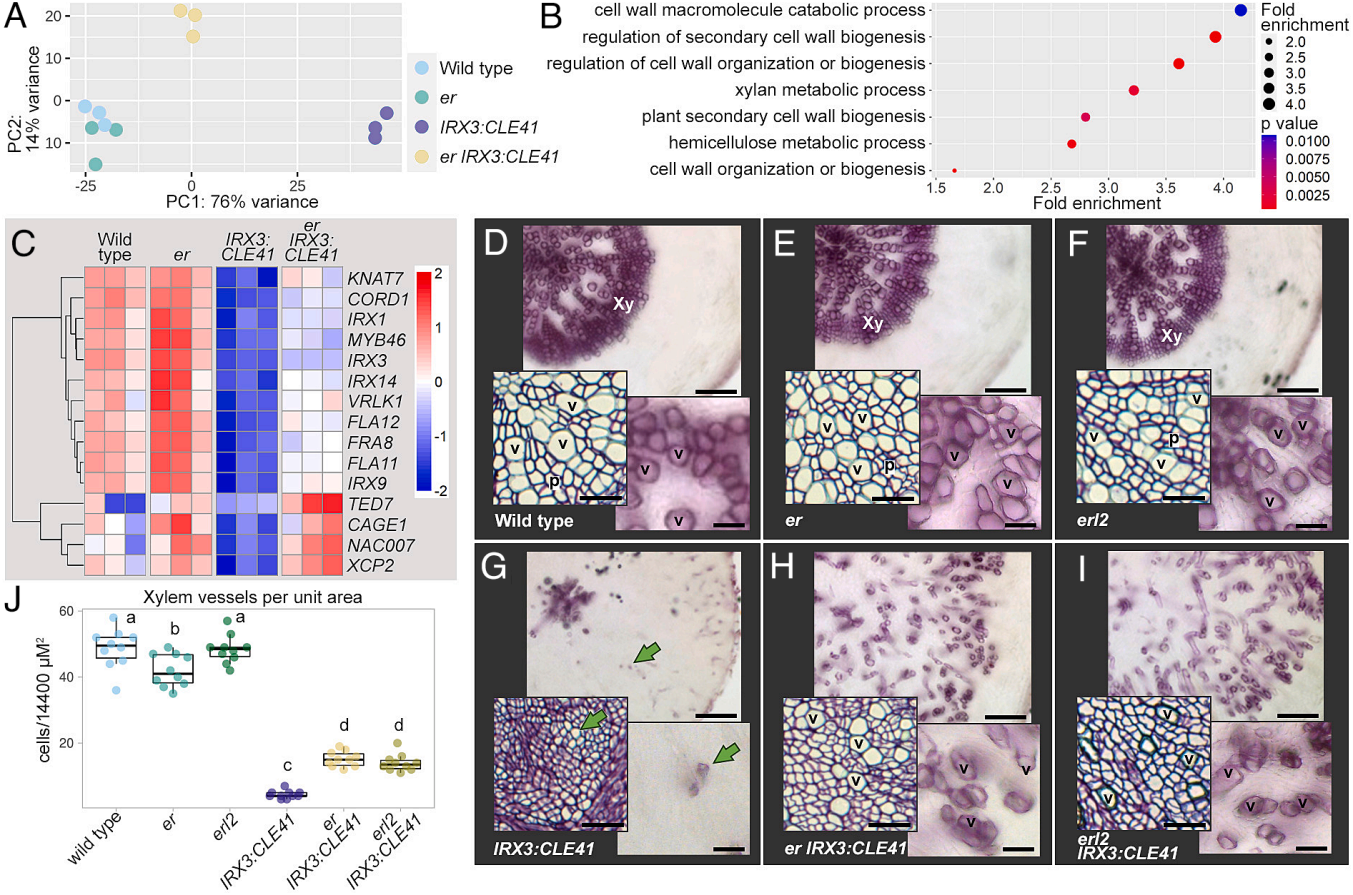
*er IRX3:CLE41* transcriptome. (*A*) Principal component analysis of wild type, *er*, *IRX3:CLE41*, and *er IRX3:CLE41* transcriptomes demonstrates that of *er IRX3:CLE41* is more similar to that of wild type and *er* than *er IRX3:CLE41*. (*B*) Biological function ontogenies enriched in *er IRX3:CLE41* transcriptomes relative to *IRX3:CLE41*. (*C*) Heatmap showing expression of select xylem-enriched genes with differential expression in *er IRX3:CLE41* relative to *IRX3:CLE41*. (*D*–*I*) Transverse sections stained with phloroglucinol (*Upper* and *Lower* *Right*) to visualize xylem differentiation with toluidine blue stained thin sections (*Lower* *Left*) for comparison. (*J*) Boxplot showing the number of xylem vessels per 14,400 µM^2^ in hypocotyl transverse sections. Letter above boxes mark significance groups (ANOVA + Tukey). Scales in *D*–*I* are 50 µM (*Upper*) or 25 µM (*Lower*); Xy is xylem, V marks vessels, green arrows point to vessel-like cells in *IRX3:CLE41*.

To better understand the processes governed by TDIF–PXY signaling to which the ER family contributes, transcriptomes of *IRX3:CLE41* and *er IRX3:CLE41* were obtained alongside wild type and *er* controls (Dataset S1A). Principal component analysis and clustering analysis were used to assess the level of similarity between the four genotypes tested. On the first principal component, which accounted for 76% of the variance between transcriptomes, *er IRX3:CLE41* was more similar to wild type and *er* than to *IRX3:CLE41*. A similar result was observed using a Spearman correlation, where again, *er IRX3:CLE41* clustered more closely with wild type and *er* than *IRX3:CLE41*, confirming earlier phenotypic analysis that *er* suppressed *IRX3:CLE41* (Fig. 5*A* and *SI Appendix*, Fig. S3*A*). Consequently, differential gene expression was explored in further detail, with a focus on expression changes between *IRX3:CLE41* and *er IRX3:CLE41* (Dataset S1B). Gene Ontology (GO) analysis of biological function supported our earlier observations which suggested that cell division was attenuated in *er IRX3:CLE41* relative the *IRX3:CLE41*, as in a pairwise comparison between these two genotypes, GO categories enriched in *IRX3:CLE41* included several associated with cell division (*SI Appendix*, Fig. S3*B* and Dataset S1C). PXY signaling has been shown to act as a positive regulator of a series of transcriptional targets in the cambium. These include the four *CAIL* cambium stem cell factors (2), *WOX4* and *WOX14* which promote vascular cell division (8, 11), and *TMO6* and *LBD4* which act redundantly with *WOX14* (10). Expression of all four *CAILS*, *WOX4, WOX14*, *TMO6*, and *LBD4* was lower in *er IRX3:CLE41* relative to *IRX3:CLE41* (*SI Appendix*, Fig. S3*C*). Thus, ER was required for the high expression of cambium cell-division promoting genes observed when TDIF levels were elevated in *IRX3:CLE41*. We then tested whether ER could regulate expression of TDIF–PXY transcriptional targets in lines without changes to TDIF–PXY signaling. An estradiol-inducible *35S:XVE>>ER* construct was generated and transformed into *er* mutants to allow conditional restoration of ER signaling. RNA-seq was performed on uninduced *er 35S:XVE>>ER*, and *er 35S:XVE>>ER* lines subjected to a 3 h estradiol treatment (Dataset S2), a timepoint consistent with capture of early ER-responsive transcripts rather than secondary targets. *WOX4* and *LBD4* both demonstrated significant increase in expression (Dataset S2C). Thus, ER can rapidly activate expression of TDIF–PXY regulated genes.

Stem cell pools are generally maintained by controlled cell division, but also by exclusion of differentiation-promoting factors. Given that PXY promotes maintenance of the cambium stem cell pool, in part by excluding xylem differentiation from those cells (3, 7), it follows that the reduction in expression of genes associated with cell division and meristem maintenance in *er IRX3:CLE41* relative to *IRX3:CLE41* may be concomitant with increases in those involved in xylem formation. A hallmark of xylem vessel formation is deposition of a large secondary cell wall which is a composite of cellulose, hemicellulose, and lignin, followed by programmed cell death (30). GO categories enriched in *er IRX3:CLE41* relative to *IRX3:CLE41* included those associated with regulation and deposition of secondary cell walls (Dataset S1D). Furthermore, 23% of genes that demonstrated a higher expression in xylem cells relative to all other cell types in a high-resolution *Arabidopsis* root single cell transcriptome (31) were up-regulated in *er IRX3:CLE41* relative to *IRX3:CLE41* representing a significant enrichment (*P* = 0.0031; Dataset S1E). Among the genes expressed at a higher level in *er IRX3:CLE41* relative to *IRX3:CLE41* were xylem differentiation regulatory genes *KNAT7*, *MYB46*, *NAC007*, and *VRLK1*; *CAGE1*, a cytoskeleton component that directs secondary cell wall deposition; enzymes that synthesize sugar polymers that constitute the cell wall, *IRX1*, *IRX3*, *IRX9*, *IRX14*, *FRA8*, *FLA11*, *FLA12*, *CORD1*; and *XCP* which promotes programmed cell death in xylem (Fig. 5*B*). As such, ER is necessary to repress xylem differentiation in *IRX3:CLE41* lines.

During vegetative growth, *Arabidopsis* hypocotyl xylem is constituted of xylem vessels and parenchyma. Lignin is deposited in secondary cell walls including those present on xylem vessels. To investigate xylem differentiation further, transverse sections were treated with phloroglucinol, which stains lignin subunits, thus marking xylem vessels. Here, striking differences were observed between *IRX3:CLE41*, *er IRX3:CLE41*, and *erl2 IRX3:CLE41* (Fig. 5 *D*–*I*). Few cells were marked by phloroglucinol stain in *IRX3:CLE41*, but when either *ER* or *ERL2* was mutated, many were apparent, albeit fewer than was present in wild type or controls. Thin sections were taken to analyze the number of xylem vessels per unit area in *er IRX3:CLE41*, and *erl2 IRX3:CLE41* relative to controls (Fig. 5). Consistent with phloroglucinol-stained tissue, these lines had more xylem elements than *IRX3:CLE41* but fewer than wild type, *er*, or *erl1* plants.

Collectively these results demonstrate that TDIF-PXY signaling pathway is attenuated in the absence of ER and ERL2. PXY–ER heteromers likely promote cambium cell division and exclude xylem differentiation in the cambium, and as such these developmental outputs are attenuated in *er IRX3:CLE41*, and *erl2 IRX3:CLE41* plants presumably as PXY–ER, PXY–ERL2, PXL1–ER, PXL1–ERL2, PXL2–ER, or PXL2–ERL2 heteromers would be unable to form.

## Discussion

TDIF–PXY signaling is central to regulation of stem cell factors in the vascular cambium. The original description of *pxy* mutants almost two decades ago included the observation that the *pxy* phenotype was stronger in the L*er* background (1), which carries an *er* mutation, and led to experiments demonstrating that *er* is an enhancer of *pxy* (11). However, the molecular mechanism underpinning that genetic enhancement has been elusive. Partial complementation of *pxy er* with cambium-expressed ER demonstrated that whatever the mechanism, it was likely to be cell-autonomous. Our FRET, split luciferase assays, and Co-IP data shown here demonstrated that members of the PXY and ER families of receptor kinases likely form complexes at the plasma membrane (Figs. 1 *D*–*J* and 2). ER and ERL2 form heteromers with all three members of the PXY family, and while ERL1 can bind to PXL1 and PXL2, there is no evidence for it interacting with PXY. Further genetic analysis, where the PXY ligand was manipulated in the presence or absence of *er* or *erl2*, demonstrated that PXY–ER heteromers influence cell division (Fig. 3) and repression of xylem differentiation (Figs. 3 and 4), two key components required for cambium maintenance. We thus propose that the interaction between PXY and ER defines ER outputs in the cambium.

It remains an interesting question for future research as to which members of the EPF ligand family are responsible for cambium regulation. Further questions surround the consequences of complex formation to signal transduction, i.e., are protein–protein interactions *required* for PXY- and ER-dependent signaling events that promote cambial cell divisions. On one hand, the genetic evidence does seem to support this. ERL1 is expressed in the cambium, but cannot form heteromers with PXY, and *erl1* does not enhance *pxy pxl1 pxl2 er* lines. By contrast, loss of *ERL2* from *pxy pxl1 pxl2 er* lines resulted in a clear phenotypic enhancement. Thus, ERL2 has a greater role in regulating cambium homeostasis, which in turn suggests that complex formation between PXY and ER family members does contribute to efficiency of signaling. On the other hand, *er* and *erl* mutant combinations do not have the characteristic intercalation of xylem and phloem observed in *pxy* lines. As such the genetic interaction is enhancement rather than epistasis, and epistasis would be expected if ER signaling was essential for all PXY outputs. Thus, either some signaling is PXY–ER complex-independent, or signaling is more efficient within the complex but still occurs without it. Nevertheless, our results demonstrate that cambium homeostasis programs collectively depend on these two receptor kinase families and their ability to form complexes. Strikingly, ANT, a cambium stem cell factor that we have shown is PXY-regulated, has been found to be phosphorylated in response to ER signaling in the cambium (27). Here, we have shown that established transcriptional targets of PXY signaling, *WOX4* and *LBD4* (8, 10), are also activated by ER-induction (Dataset S2C).

ER has previously been shown to bind to the adaptor protein SOBIR1 to control the rate of differentiation in xylem cells (15), and with coreceptor TOO MANY MOUTHS to define epidermal patterning (19, 21), thus ER receptor heterodimerization with different tissue specific partners defines specific ER outputs within those tissues. In vascular tissue, there is abutment of cambium tissue containing PXY–ER complexes and xylem, containing SOBIR1-ER heteromers (15). One explanation is that the arrangement of different ER heteromers in adjacent domains collectively contributes to vascular tissue formation and patterning. An increase in radial growth was observed in *er erl1* lines, due to a failure to activate xylem differentiation programs, and this further illustrates why ER family signaling outputs require cell type–specific programs. While there are many examples of coreceptor pairs and binding of receptors to adaptor proteins, there are very few examples of receptor–receptor interactions where the two proteins in complex are activated by different ligands, such as that between PXY and ER. A complex containing CLAVATA1 and ACR4 receptor proteins regulating root stem cells may be a notable example (32) should ligand binding be established for ACR4. In metazoans, receptor–receptor interactions have been described for different B-type receptors of the neurotransmitter GABA (33–35), and epidermal growth factor receptor, which plays roles in cell proliferation and migration and has been demonstrated to form receptor–receptor complexes with both receptor kinase NOK/STYK1 (36), and with platelet-derived growth factor receptor β (37).

Previously, we described loss of secondary growth in *pxy pxl1 pxl2 er erl1 erl2* lines, demonstrating a genetic interaction between members of the PXY and ER receptor families. Here, we have shown that this genetic interaction is likely underpinned by formation of PXY–ER receptor–receptor complexes.

## Materials and Methods

### Plant Materials and Growth Conditions.

*Arabidopsis thaliana* (Columbia ecotype) plants were grown at 22 °C on a 16 h/8 h light/dark cycle. Seeds were surface-sterilized with absolute ethyl alcohol for 5 min and 75% ethanol for 15 min, then washed three-four times with sterilized distilled water under aseptic conditions. Surface-sterilized seeds were germinated on half-strength Murashige and Skoog (MS) medium in an incubator. After 7 d, the seedlings were transferred into soil. Plants for analysis were grown in a drop and grow container farm (LettUs Grow).

*pxy*, *er*, *pxy er*, *pxy pxl1 pxl2*, *pxy pxl1 pxl2 er*, *pxy pxl1 pxl2 er erl2*, *pxy pxl1 pxl2 er erl1* and *IRX3:CLE41* lines have been described previously (6, 11, 12, 17). *er IRX3:CLE41*, *erl1 IRX3:CLE41*, and *erl2 IRX3:CLE41* were generated by crossing and selected in F2 and F3 populations on the basis of phenotype, PCR genotyping, and plant resistance. *pxy er PXY:ER* lines were generated by transforming *pxy er* plants with binary vector pGGZ003 carrying a *PXY* promoter-*ER* gene body fusion generated using Greengate. Homozygous lines were selected in the T3 generation. *35S:XVE>>ER* lines were generated by transforming WT and *er* with binary vector pMDC7 carrying ER cDNA generated using gateway. Homozygous lines were selected in the T3 generation. For Estradiol treatment, a 5 mM working concentration of 17-β-estradiol (17-β, Sigma) was used for XVE-based gene induction. Estradiol treatment was performed by transferring plants to 0.5 × MS (without sugar) plates supplied with 5 mM 17-β-estradiol and continuing growth for the indicated time.

*Nicotiana benthamiana* seeds were germinated on soil and grown at 24 °C in a 12 h/1 h light/dark cycle.

### Binary Vectors for Protein Interaction Studies.

For FRET and Co-IP, primers were designed against *A. thaliana PXY*, *PXL1*, *PXL2*, *ER*, *ERL1*, and *ERL2* and used to amplify sequences from cDNA. Amplified cDNAs were subsequently fused with CFP/YFP/HA/FLAG tags via Infusion and cloned into pGWB502 using LR clonase II to generate constitutive expression vectors.

For split luciferase vectors, the cDNAs of *PXY*, *PXL1*, *PXL2*, *PXY-ECD/PXL1-CD* (ECD Extracellular domain; CD Cytoplasmic domain), *ER*, *ERL1*, *ERL2, ER-ECD/ERL1-CD, ERL1-ECD/ER-CD*, *ER*, *ERL1*, *ERL2* were cloned into pGWB-nLUC /pGWB-cLUC using gateway to generate constitutive expression vectors.

For insect cell protein expression pBV-plasmids with ER-twinstrep, PXY-flag, or bicistronic PXY-flag/ER-twinstrep expression constructs were ordered from Vector Builder and sequenced.

### FRET.

Recombinant vectors *35S:PXY-CFP*, *35S:ER-YFP*, *35S:ERL1-YFP,* and *35S:ERL2-YFP* were used for FRET. Vectors were transformed into tobacco leaf cells using *Agrobacterium tumefaciens* strain GV3101. 5 mL of *Agrobacterium tumefaciens* strain GV3101 harboring a binary vector was grown to an OD_600_ of 0.6, collected by centrifugation in 15 mL tubes, washed twice, and resuspended in 1 mL infiltration buffer (1 M pH 5.7 MES 2.5 mL, 0.5 M D-Glucose 2.8 mL, 0.05 M Na_3_PO_4_ 12H_2_O 2 mL, 200 mM Acetosyringone 25 μL, with distilled water added to make a total volume of 50 mL) at room temperature. The final OD_600_ fell within the range of 0.01 to 0.1. Cells from various constructs and P19 (38) were combined in a ratio of 1:1:1 (v/v/v). Transfected tobacco leaves were incubated in a greenhouse (16 h/8 h light/dark) at 24 °C for a minimum of 48 h.

FRET imaging and quantification was carried out on a Zeiss LSM880 confocal microscope. FRET images were acquired in normal channel imaging mode using two GaAsP detectors and a Zeiss Objective Plan-Apochromat 63×/1.4 Oil DIC M27 (420782-9900-799) objective lens. A 458 nm laser was used to excite the CFP/FRET detection and a 514 nm laser was used to excite the YFP. Detector 1 (Ch1) had an emission window set to 433-501 nm for CFP. Detector 2 (Ch2) had an emission window set to 500 to 566 nm for imaging the FRET/YFP. Calculation of FRET efficiencies used Lambda mode with the spectral detector set to a range of 409 to 607 nm and the 458 nm laser used as excitation source and a W Plan-Apochromat 40×/1.0 DIC M27 (421462-9900-799) objective lens. Scans were taken of CFP–YFP pairs and CFP only (that gives values of Donor contribution to the FRET channel). After acquiring a spectral scan in Zen software, regions of interest (ROIs, at the presumed location of the plasma membrane of a transformed cell) were selected within the Spectral Unmixing tool. Values corresponding to the peaks of emission for CFP and YFP were used for subsequent FRET calculations. FRET efficiency was calculated using the formula: FRET Efficiency = Fa[minus the CFP contribution]/(Fd+Fa[minus the CFP contribution]).

### Split Luciferase Assay.

Split luciferase assay was performed using *Nicotiana* leaves infiltrated with recombinant vectors *35S:nLUC, 35S:PXY-nLUC*, *35S:PXL1-nLUC*, *35S:PXL2-nLUC*, *35S:PXY-ECD/PXL1-CD-nLUC*, *35S:ER-cLUC, 35S:ERL1-cLUC*, *35S:ERl2-cLUC, 35S:ER-ECD/ERL1-CD-cLUC,* and *35S:ERL1-ECD/ER-CD-cLUC*. Cells carrying *n* and *c* constructs and P19 (38) were combined in a ratio of 1:1:1 (v/v/v). Transfected tobacco leaves were incubated in a greenhouse (16 h/8 h light/dark) at 24 °C for a minimum of 48 h. After incubation, the infiltrated leaves were transferred to 1/2 MS medium and immediately drops of 1 mM d-luciferin in 1/2 MS liquid medium were applied on the surface of leaves for 5 min. The samples were then placed in a dark box (Photometric, model: LMZ-DRK-BOX), and luminescence was captured every 2 min using a Photometrics Evolve 512 EMCCD camera (Photometric, model: Evolve® 512, catalog number: EVO‐512‐M‐FW-16-AC-RP) equipped with a 17-mm fixed lens/0.95 (Edmund Optics, model: 59-832) and an additional 125-mm lens (Thorlabs, model: LA1384-A). The EMCCD multiplier gain was 1,000 and the exposure time was 120 s.

### Co-IP In Planta.

Co-IP was performed using either *Nicotiana* leaves infiltrated with *Agrobacterium* harboring plant expression vectors, or *Arabidopsis* protoplasts. Protoplasts were isolated from root cell suspension cultures (39) and then transfected with 10 µg plasmid DNA of the appropriate vectors and overnight dark incubated at room temperature in glucose-mannitol (GM) medium). Infiltrated *Nicotiana* leaves were incubated in a greenhouse (16 h/8 h light/dark) at 24 °C for a minimum of 48 h. Protoplasts or tobacco leaves were treated with 10 µM MG132 (in GM/infiltration buffer) 6 h prior to subsequent processing. The protoplast cells were centrifuged at 1,200 rpm for 7 min the next day and supernatant was removed. Tissue was finely ground into a powder using mortar and pestles. Lysis binding buffer (50 mM Tris-HCl, pH 7.6, 150 mM NaCl, 1% NP-40, 0.1% SDS, 1 mM PMSF, and Protease Inhibitor Cocktail—EDTA-Free) was added to the samples with a sample to buffer ratio between 1:1.5 and 1:2 (w/v). The resulting sample lysates were incubated on a shaker at 4 °C for 1 h and centrifuged for 20 min at maximum speed and 4 °C.

Meanwhile, 30 to 40 μL of either Anti-DYKDDDDK or Anti-HA beads were placed on a magnetic rack, storage buffer removed, and washing buffer (consisting of 50 mM Tris-HCl, pH 7.6, 150 mM NaCl, 1% NP-40, 0.1% SDS, and 1 mM PMSF) added. Beads to washing buffer ratio was maintained at 1:10 (v/v) though two further washes. 300 to 400 μL of sample lysate was introduced to the beads and incubated on a rocking table at 4 °C for 1 to 2 h. Beads were washed 3 to 4 times as described earlier. Beads and 50 to 60 μL SDS-loading buffer were boiled at 94 °C for 5 min and then placed on a magnetic rack. Supernatants were subjected to western blots using standard methods.

### Sf21 Cell Culture, Protein Expression and Co-IP.

*Sf21* cells were maintained in serum-free SFX medium (Cytiva) at 27 °C, 100 rpm. The cells were passaged every 48 to 72 h to maintain a cell density between 0.5 × 10^6^ and 4 × 10^6^. Proteins were produced using the Bac-to-Bac system in *Sf21* insect cells based on protocol by Ciccarone et al. (40). Briefly, to produce baculovirus vectors the pBV Baculovirus expression plasmid containing expression ER-twinstrep, PXY-flag or PXY-flag/ER-twinstrep constructs were transformed into competent DH10Bac *Escherichia coli* cells and incubated for 48 h at 37 °C. Formation of competent expression construct bacmid colony was detected using x-gal, lac-z system. After overnight expansion isopropanol purified bacmid was transfected to sf21 insect cells using Fugene 6 (Promega). After 96 h, transfected cells were collected and used as V0 viral stock and added to insect cells 1:100 ratio (1 × 10^6 cells/mL). Baculovirus stocks were expanded twice similarly, this v2 stock was then added in 1:20 ratio to insect cell culture (1 × 10^6 cells/mL) for protein production and collected after 96 h.

To isolate total cellular membranes, cells were pelleted at 800 × g and washed with cold PBS. The cell pellet was resuspended in hypotonic buffer [20 mM HEPES pH 7.5, 1 mM DTT, protease, and phosphatase inhibitors (Roche)] for 20 min on ice. After adjusting to 150 mM NaCl and 1 mM MgCl^2^, cells were disrupted using a microfluidizer (15,000 psi, 10 min) and clarified at 1,000 × g. Membranes were isolated by ultracentrifugation at 45,000 rpm for 1 h at 4 °C and resuspended in solubilization buffer [50 mM Tris pH 8, 100 mM KOAc, 250 mM Sucrose, 2 mM Mg(OAc)_2_, 1× protease inhibitor (Roche)]. Membrane fractions enriched for PXY/ER or ER were solubilized using a solubilization buffer (50 mM Tris-HCl pH 7.5, 150 mM NaCl, 2% DDM, and protease inhibitor cocktail). Samples were kept on ice for 15 min after which they were centrifuged at maximum speed for 10 min at 4 °C and diluted to a final DDM concentration of 0.1%. An aliquot of the diluted supernatant was reserved for SDS–PAGE analysis. Anti-FLAG M2 magnetic beads (Sigma, M8823) were washed four times with a wash buffer (50 mM Tris-HCl pH 7.5, 150 mM NaCl, 0.05% DDM). Anti-Flag M2 magnetic (25 μL) beads were used per reaction. Diluted lysates were incubated with the washed beads for 2 h at 4 °C with end-over-end rotation. FLAG-tagged proteins were eluted by incubating beads with 50 μL of 3× FLAG peptide solution (200 µg/mL) for 30 min at 4 °C. PXY-flag recombinant protein traveling in SDS-page in western blot exhibited extensive streaking and splitting to smaller bands. Total protein load to SDS-page gel was adjusted accordingly.

### Plant Anatomy.

For thin sections stained with toluidine blue, plant material was fixed in FAA overnight and dehydrated through an ethanol series prior to embedding in JB4 (polysciences) according to the manufacturer’s instructions. Briefly, dehydrated samples were subjected to successive ethanol:JB4 infiltration solution 75%:25%, 50%:50%, 25%:75% solutions followed by 2 overnight incubations in 100% JB4 infiltration solution. Samples were dried on tissue, placed in molds to which embedding solution was added. Blocks were covered with parafilm and hardened overnight. 4 μM sections were taken on a rotary microtome fitted with a glass knife, which were mounted on glass slides, stained with 0.025% aqueous toluidine blue and mounted with histomount.

Lignin was stained with phloroglucinol. Fresh sections embedded in 4% Agarose were taken using a vibratome which were incubated in phloroglucinol-HCl solution for 30 min prior to mounting in glycerol for imaging.

### Gene Expression.

For *pER:gER-tagRFP* cloning, 2 Kb promoter and full-length *ER* genomic DNA without stop codon were amplified with primers pER-F+pER-R (ggggacaactttgtatagaaaagttgcaagatcctggatttgtaactga; ggggactgcttttttgtacaaacttgt tctcacacacagtcttaaaacga) and gER-F+gER-R (ggggacaagtttgtacaaaaaagcaggctc gatggctctgtttagagatattgttctt; ggggaccactttgtacaagaaagctgggtgctcactgttc tgagaaa taacttgtcc), respectively. The PCR products were recombined into MultiSite Gateway compatible pDONR entry vectors using a BP clonase reaction resulting in 1R4z-pER and 221z-gER. The *pER:gER-tagRFP* binary vector was generated by combining 1R4z-pER, 221z-gER, and 2R3a-tagRFP-OcsT (41) and destination vector pFGm43GW (42) in a single MultiSite Gateway LR reaction. To generate the *pER:gER-tagRFP* and *pPXY:gPXY-YFP* double reporter, *pER:gER-tagRFP* construct was transformed into *pPXY:gPXY-YFP*;*pxy* (2) background using a floral dipping method (43). The positive T1 transformants were selected based on the seed-specific GFP fluorescence. To analyze the *pER:gER-tagRFP*; *pPXY:gPXY-YFP* double reporter, 20 T1 transformants were analyzed. For cross sections of hypocotyl, samples were fixed as previously described (2), prior to embedding in 5% agarose. Agarose blocks were cut with the vibratome into 200 µm sections for confocal analysis. Sections were placed in 1×PBS and stained with SR2200 for cell wall staining. Confocal imaging was performed on PBS-mounted samples with a Stellaris 8 confocal microscope. Samples were imaged in the sequential scan mode.

For RNA-seq *ER* induction, WT and *35S:XVE>>ER* seeds were first germinated on 0.5 × MS plates for 7 d, and then the 7-d-old seedlings were transferred to 5 mM 17-β induction or mock-plates for 3 h. For each sample, 5-6 seedlings were collected for RNA prep. Three or four biological repeats were conducted for each time point. For *er IRX3:CLE41* lines and controls, RNA was isolated from 5-wk-old *Arabidopsis* hypocotyls in biological triplicate using ReliaPrep™ RNA Miniprep and subjected to RNA-seq on the Illumina platform (Novogene). 150 base paired end reads were mapped to the *Arabidopsis* TAIR10 genome (EnsemblePlants, release 58) sequence with corresponding gtf file to obtain reads per gene using STAR aligner (v 2.7.11a) (44). Read count per gene was analyzed using DESeq2(v 1.40.2) (45) to get *P* values, adjusted *P* values, and log2 fold changes. Sample PCA (principal component analysis) plot was generated using the plotPCA function of DESeq2 after variance stabilization transformation. Sample correlation heatmap and gene expression heatmap were generated using pheatmap(v1.0.12). GO (gene ontology) analyses were performed using PANTHER (46) and the results were plotted using ggplot2(v3.4.4). The raw sequencing datasets are available on the NCBI Gene Expression Omnibus (GEO) server under accession numbers GSE263680 (*er IRX3:CLE41* and controls) (47), and GSE310491 (*ER* induction and controls) (48).

## Supplementary Material

Appendix 01 (PDF)

Dataset S01 (XLSX)

Dataset S02 (XLSX)

## Data Availability

RNA-seq data have been deposited in GEO/ENA [GSE263680 (47) GSE310491 (48)]. The stable accession numbers of the genes described in this article are PXY (At5g61480), PXL1 (At1g08590), PXL2 (At4g28650), ER (At2G26330), ERL1 (At5g62230), ERL2 (At5g07180), CLE41 (At3g24770), ANT (At4g37750), AIL5/PLT5 (At5g57390), AIL6/PLT3 (At5g10510), AIL7/PLT7 (At5g65510) WOX4 (At1g46480), WOX14 (At1g20700), TMO6 (At5g60200), LBD4 (At1g31320), BES1 (At1g19350), BZR1 (At1g75080), KNAT7 (At1g62990), CORD1 (At3g14170)), IRX1 (At4g18780), MYB46 (At5g12870), IRX3 (At5g17420), IRX14 (At4g36890), VRLK1 (At1g79620), FLA12 (At5g60490), FRA8 (At2g28110), FLA11 (At5g03170), IRX9 (At2g37090), TED7 (At5g48920), CAGE1 (At1g05170), NAC007 (At1g12260), XCP2 (At1g20850) (49). All other data are included in the manuscript and/or supporting information.
