# Supplementary material for: A receptor kinase complex refines cambium activity in Arabidopsis
Source: Proc Natl Acad Sci U S A. 2026 Jun 22;123(26):e2532481123. doi: 10.1073/pnas.2532481123 (PMC13321232; doi:10.1073/pnas.2532481123)
Supplement: Supplementary file 1 — Appendix 01 (PDF) [file pnas.2532481123.sapp.pdf]

PNAS

Supporting Information for
A receptor kinase complex refines cambium activity in *Arabidopsis*

Qing He, Hanan Alhowty, Prodeep Paudel, Xixi Zhang, Wenbin Wei, Tuomas Sipilä, Ehmke Pohl, Ari Pekka Mähönen, Ville O Paavilainen, Raymond Wightman, Yuan Qin, J Peter Etchells

Peter Etchells
Email: Peter.Etchells@durham.ac.uk

Yuan Qin
Email: yuanqin@fafu.edu.cn

This PDF file includes:

Figures S1 to S5
Legends for Datasets S1 to S2

Other supporting materials for this manuscript include the following:

Datasets S1 to S2

Supporting Information Figures

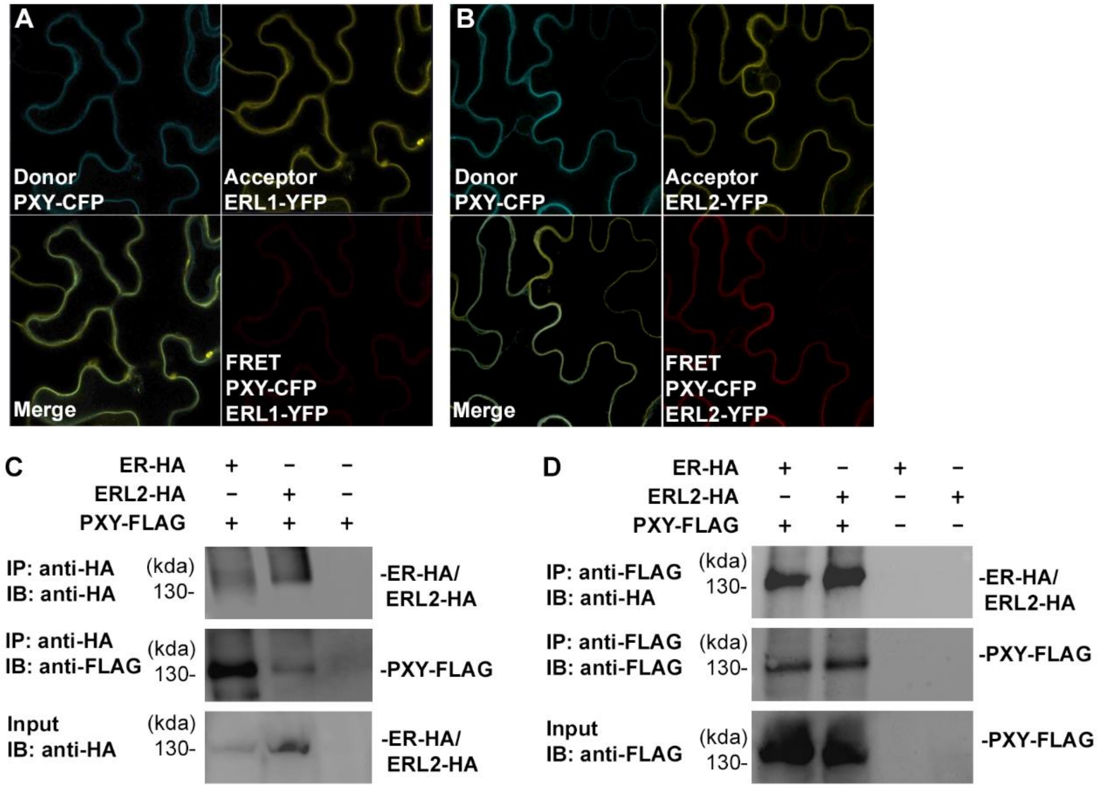

Fig. S1. PXY interacts with ERL2 in *Nicotiana* leaves.

(A-B) FRET occurs between PXY-CFP and ERL2-YFP but not between PXY-CFP and ERL1-YFP. Localisation of PXY-CFP, ERL1-YFP, overlap between the two, and an absence of FRET signal (A). Localisation of PXY-CFP, ERL2-YFP, overlap between the two, and FRET signal (B). **(C-D)** Coimmunoprecipitation of PXY-FLAG with ER-HA or ERL2-HA and *vice versa* in *Nicotiana* epidermis. Immunoblot (IB); Immunoprecipitation (IP).

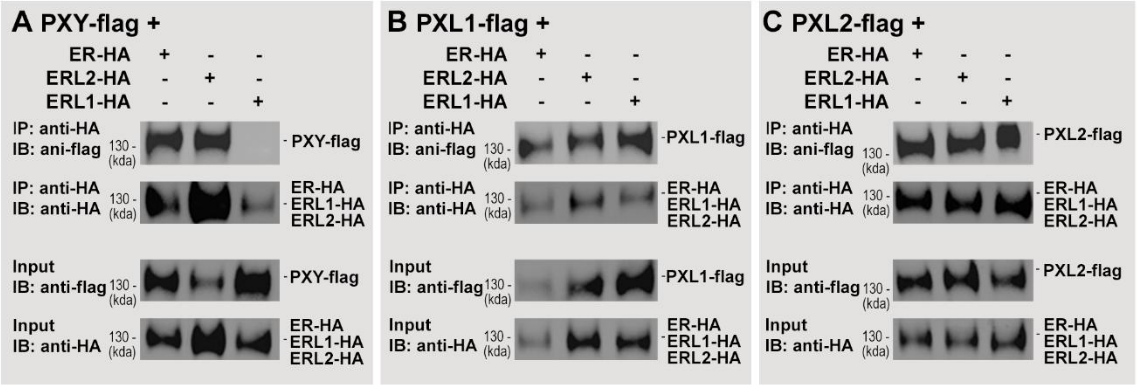

Fig. S2. PXY interacts with ERL2 in *Arabidopsis* cells

Coimmunoprecipitation of PXY-flag **(A)**, PXL1-flag **(B)**, or PXL2-flag **(C)**, with ER-HA, ERL1-HA and ERL2-HA. Immunoblot (IB); Immunoprecipitation (IP).

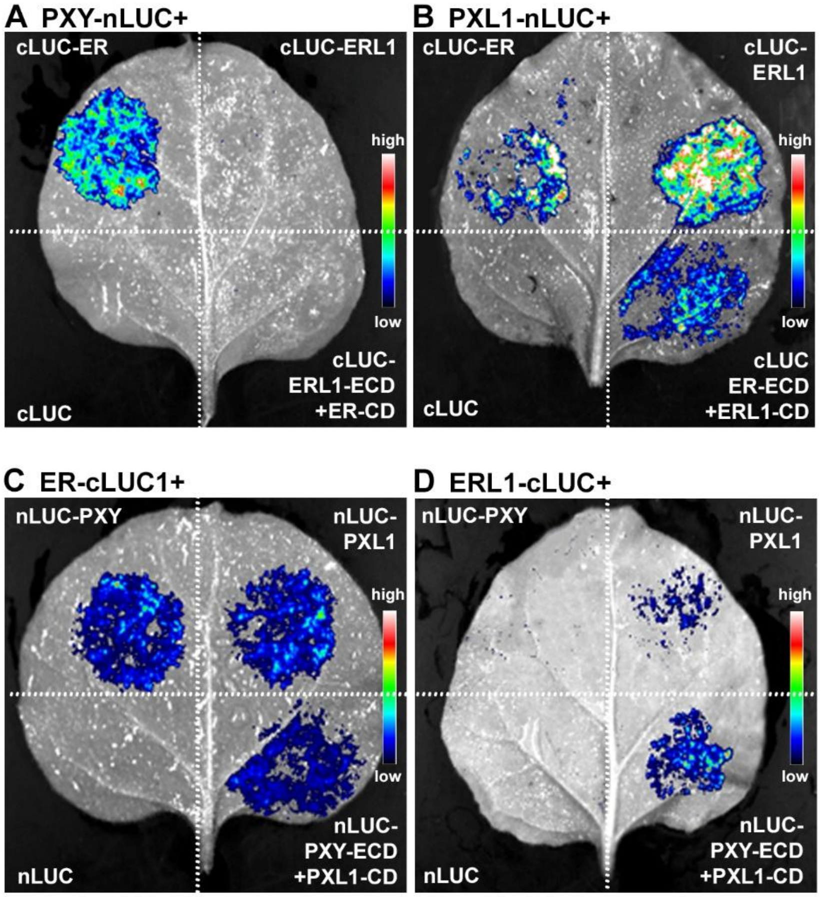

Fig. S3. Interactions between chimeric proteins

(A-B) Split luciferase assays in *Nicotiana* leaves in which the n-term of Luciferase was fused to PXY (A) or PXL1 (B) and was co-infiltrated with the c-term alone, or the c-term fused to a chimera containing the ERL1 extracellular domain and the ER cytoplasmic domain. **(C-D)** Split luciferase assays in *Nicotiana* leaves where the c-term of Luciferase was fused to ER (C) or ERL1 (D) and was co-infiltrated with the n-term alone, PXL1, or a chimera containing the PXY extracellular domain and the PXL1 cytoplasmic domain.

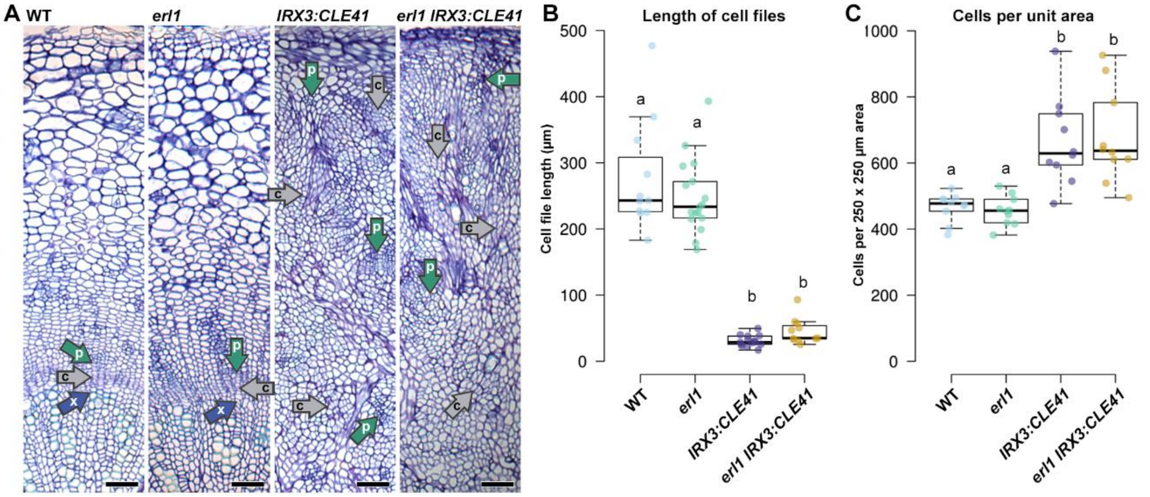

Figure S4. ***erl1*** **cannot supress TDIF over-production phenotypes**

(A) Transverse sections of hypocotyls. Thin sections show the extent of radial growth with grey arrows marking cambium, green arrows marking phloem, and blue marking xylem. Xylem and phloem are separated by cambium in wild type and *erl1* but in *IRX3:CLE41* and *erl1 IRX3:CLE41* lines few xylem cells are apparent, and the cambium is disordered. **(B)** Boxplot showing length of cell files running parallel to the radial axis of the stem. **(C)** Boxplot showing cells in a 250 x 250 µm area in hypocotyl transverse sections. Letter above boxes mark significance groups (B and C; ANOVA + Tukey). Scales are 50 µM; p is phloem, c cambium, and x xylem.

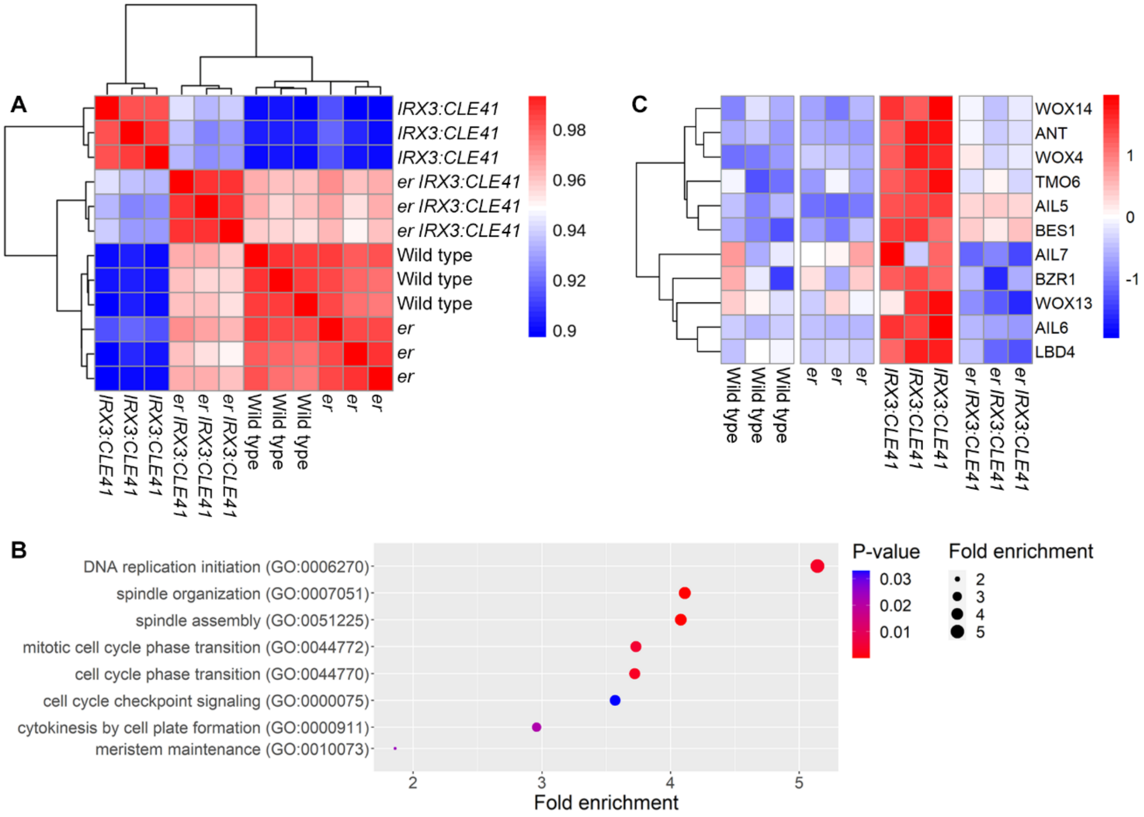

Figure S5. Analysis of *er IRX3:CLE41* transcriptome

(A) Cluster analysis of wild type, *er*, *IRX3:CLE41* and *er IRX3:CLE41* transcriptomes demonstrates that of *er IRX3:CLE41* is more similar to that of wild type and *er* than *er IRX3:CLE41*. **(B)** Biological function ontogenies associated with cell division enriched in *er IRX3:CLE41* transcriptomes relative to *IRX3:CLE41*. **(C)** Heatmap showing expression of transcriptional targets of TDIF-PXY signalling in *er IRX3:CLE41* relative to *IRX3:CLE41*.

Dataset S1 (separate file). Transcriptome data and analysis from WT, *er*, *IRX3:CLE41*, and *er IRX3:CLE41* lines.

Dataset S2 (separate file). Transcriptome data and analysis from WT, *er 35S:XVE>>ER* (uninduced), and *er 35S:XVE>>ER* (3h induced) lines.
